# Transformation of Endophytic *Bipolaris* spp. Into Biotrophic Pathogen Under Auxin Cross-Talk With Brassinosteroids and Abscisic Acid

**DOI:** 10.3389/fbioe.2021.657635

**Published:** 2021-07-28

**Authors:** Muhammad Junaid Yousaf, Anwar Hussain, Muhammad Hamayun, Amjad Iqbal, Muhammad Irshad, Ho-Youn Kim, In-Jung Lee

**Affiliations:** ^1^Department of Botany, Abdul Wali Khan University Mardan, Mardan, Pakistan; ^2^Department of Food Science and Technology, Abdul Wali Khan University Mardan, Mardan, Pakistan; ^3^Smart Farm Research Center, Korea Institute of Science and Technology (KIST), Gangneung, South Korea; ^4^Department of Applied Biosciences, Kyungpook National University, Daegu, South Korea

**Keywords:** symbiosis, phytohormones, yucasin, *Zea mays*, biotroph

## Abstract

Auxin is the reciprocal signaling molecule, which interferes with other phyto-hormonal and physiological processes during plant–microbes interaction. In this regard, *Bipolaris* spp., a growth-promoting endophytic fungus was used to inoculate pre-stressed *Zea mays* seedlings with yucasin (IAA inhibitor). The IAA-deficient host was heavily colonized by the endophyte that subsequently promoted the host growth and elevated the IAA levels with a peak value at 72 h. However, the seedling growth was inhibited later (i.e., at 120 h) due to the high levels of IAA that interfered with the activity of phytoalexins and brassinosteroids. Such interference also modulated the endophytic fungus from symbiotic to biotrophic pathogen that left the host plants defenseless.

## Introduction

Auxin has long been documented to elicit various physiological and developmental processes in plants. Perhaps, it is a less-known fact that auxins produced by the plant pathogenic microbes have dual impact on plant physiological aspects (Ludwig-Müller, 2015). For example, at the plant wound sites, auxins produced by the microbes results in the formation of galls and tumors (Mashiguchi et al., 2018). Plants use multiple pathways to synthesize auxins. One of the well-established auxin biosynthesis pathway is the indole-3-pyruvic acid (IPA) pathway, regulated by the tryptophan aminotransferase of Arabidopsis 1/tryptophan aminotransferase-related proteins (TAA1/TARs) and flavin-containing monooxygenases of the YUCCA family (YUCCA) (Brumos et al., 2018). This is a major pathway for production of free IAA in plants (Kasahara, 2016). TAA1 catalyses the conversion of tryptophan (Trp) to IPA and then YUC involves in oxidative decarboxylation of IPA to form IAA through IPA pathway. Genetic studies revealed that YUC can act as a limiting rate enzyme in the biosynthesis of IAA. Therefore, YUC is regarded as a crucial enzyme in the regulation of many physiological and developmental processes of plants (Brumos et al., 2018).

Moreover, yucasin has been noticed as an effective inhibitor of YUC, as it significantly reduced endogenous IAA in maize and suppressed high-auxin phenotypes in *Arabidopsis* (McClerklin et al., 2018). A high level of endogenous auxins (from normal physiological concentration) promotes susceptibility in *Arabidopsis* irrespective to salicylic acid (SA)-mediated defense (Mashiguchi et al., 2018). Salicylic acid signaling is an integral part of plant immune response to pathogens mediating both pathogen-triggered immunity (PTI) and effector-triggered immunity (ETI). Accumulation of SA activates expression of pathogenesis-related (PR) genes, making the host plant resistant to bacterial microbial pathogens (Miao et al., 2019). Plants over-expressing SA mostly exhibit the phenotypes that are indicative of auxin-deficient mutants, suggesting that SA interferes with auxin responses (Vidhyasekaran, 2016). Auxins cross-talk with other phytohormones is an important phenomenon during plant growth and development and also in shaping plant interactions with microbes (Meena et al., 2017). For instance, auxin can inhibit cytokinin not only on its biosynthesis but also on its signaling (Naseem and Dandekar, 2012). Besides antagonism between auxin and cytokinin, synergism between these two hormones is also important in several developmental processes. During Arabidopsis interaction with *Pseudomonas syringae*, auxin accumulates in the cell, which represses the SA pathway making the host susceptible to the path (Argueso et al., 2012). In this interaction, auxin may repress responses to cytokinin by keeping the type-A ARRs activated, which are negative regulators of cytokinin perception in plants of cytokinin signaling (Argueso et al., 2012).

Similar to auxins, brassinosteroids (BRs) and abscisic acid also produce various physiological responses in plants, such as cell elongation and division, seed development and dormancy (Vidhyasekaran, 2016). IAA can establish a cross-talk with BR by interacting with BZR1 and PIF4 genes, inducing a hypocotyl elongation. IAA can also start a negative cross-talk with ABA at seed and bud dormancy (González et al., 2017). Likewise, IAA can interfere with the biosynthesis of other secondary metabolites during plant growth and development. Presently, we have adopted a similar approach to analyze the antagonizing effect of IAA (at minimal, adequate, optimal, and excessive concentrations) on phytohormonal and growth responses during plant–microbe interactions by using maize–*Bipolaris* system. We have used IAA inhibitor to cut the production of IAA by inhibiting its major biosynthesis pathway and noted responses of the seedlings. Gene expression data available in public database has also been utilized and analyzed to look at the broader picture. This study will help to further elaborate the role of IAA in plant immunity against pathogens under cross-talks with other hormones.

## Materials and Methods

### Plant Materials

*Zea mays (var. Hysun-33)* seeds were obtained from the local market and stored at 4°C for 21 days prior to the experiment. The seeds were dipped in 0.1% of HgCl_2_ for 10 s and then primed in 0.05 mM KCl solution for 30 s. The primed seeds were finally rinsed with sterilized distilled water to wash away the traces of KCl. The seeds were subsequently sterilized with 70% of ethanol for 30 s and washed 4 times with autoclaved distilled water. Afterward, the sterilized primed seeds were placed on double-layered sterilized wet filter papers in Petri plates. All the Petri plates were wrapped in an aluminum foil and incubated at 25°C for 7 days. Germinated seeds were then shifted to pots having a Hoagland solution (HG). The HG was prepared using the standard protocol (Hoagland and Arnon, 1950). After staying in HG for 3 days, the leaves of the seedlings were first sprayed with 10 mM yucasin (Y) and then inoculated with *Bipolaris* (B) after 12 h of the stress (Y–B). The seedlings labeled as “B” did not receive yucasin treatment.

The seedling growth conditions were determined by analyzing the relative growth rate (RGR) and the net assimilation rate (NAR). The RGR was analyzed by taking RGR = (1/W) (dW/dt) in which dW is the increase in dry weight per increase in time (dt) divided by existing biomass (W) (Rezvani-Moghaddam, 2020). However, NAR was obtained by using the formula (1/L_A_) (dW/dt) wherein L_A_ represents the leaf area divided on dW/dt (ratio of dry weight increase to change in time) (Moualeu-Ngangue et al., 2017). RGR was expressed in g/g/day while NAR was expressed in percent change after each time interval.

### Preparation of Fungal Inoculum

The fungal strain *Bipolaris* CSL1, isolated and identified previously in our laboratory (Asaf et al., 2019) was maintained on potato dextrose agar (PDA). Fresh culture grown on PDA at 28°C was used as a source of fungal spores. Fungal spores (0.02 g) were carefully taken in 250 mL of potato dextrose broth and kept in shaking incubator operated at 2,400 rcf and 25°C for 5 days. Inoculum preparation was carried out by taking 1 mL of the spore suspension on day 5 of the incubation and transferred to a fresh potato dextrose broth, which was then kept overnight in shaking incubator at 25°C. The optical density of the overnight spore suspension was adjusted to 0.02 at 600 NM with the help of autoclaved distilled water.

### Determination of Fungal Root Colonization

Maize seedlings were exposed and inoculated with *Bipolaris* and yucasin at 24-h interval. The seedlings along with root segments were placed on a Petri plate containing PDA media and incubated at 25°C. The relative number of the root segments occupied by the fungus was determined. Colonization was expressed in the form of percent colonization, calculated as:

Number of root segments occupied by fungus/total number of segments plated × 100.

### Preparation of Sample

Maize leaves were ground into a fine powder in the presence of liquid nitrogen. The ground powder (2 g) was then mixed with 200 mL of methanol and kept in soxhlet apparatus for 36 h at room temperature. The extracts so obtained were first filtered and then concentrated under reduced pressure in a rotary evaporator. Finally, the concentrated extract was evaluated for the presence of secondary metabolites, enzymatic activities, phytohormones, and radical scavenging activities. On the other hand, the root exudates (RE) were simply filtered through a fine filter paper and subjected to the determination of the above-mentioned phytochemicals.

### Electrolytic Leakage

Electrolytic leakage (EL) was measured as described by Lutts et al. (2004). Briefly, 0.3 g of leaf from every individual plant was washed with deionized water and then placed in 15 mL of falcon tube containing 15 mL of deionized water. The samples were incubated for 2 h at 25°C. The electrolytic conductivity of the sample (L_1_) was then recorded. Samples were then autoclaved at 120°C for 20 min, cooled down to 25°C and the EC (L_2_) was measured again.

The final reading was obtained using the formula:

$$\begin{array}{l} EL\;\left(\%\right)=\frac{L_{1}}{L_{2}}\times 100 \end{array}$$

### Secondary Metabolite Determination

#### Proline Determination

The sample (1 mL) was homogenized in 4 ml of 3% sulfo-salicylic acid and centrifuged at 6,077 rcf for 5 min. Acidic ninhydrin reagent (2 mL) was then added to the mixture and vigorously shaken. Ninhydrin reagent was made by heating 1.25 g of ninhydrin in 20 mL of 6 M phosphoric acid and 30 mL of pure glacial acetic acid. The sample was heated at 100°C for 1 h and the resulting pellet was dispensed in 4 mL of toluene. ODs were recorded at 520 nm using UV–vis spectrophotometer (Lambda 1050).

#### Total Phytoalexins Determination

Colorimetric determination of total phytoalexins was carried out by adding Mayer's reagent and 80% ethanol to 2 mL of the samples and the ODs were recorded at 400 nm using UV–vis spectrophotometer (Lambda 1050).

#### Hormonal Determination

##### IAA Analysis

The leaf and root extracts so obtained were filtered through a 0.2-μm membrane using the filtration syringe system. Samples (20 μL) were injected into a reverse phase C18 HPLC column, and elution was carried out with a mixture of 35% methanol (in %1 acetic acid) using isocratic pump. The eluate was monitored at 254 nm. Standard IAA was used to identify the desired peak in the samples. Method validation was performed by adding known quantities of the standard IAA (10 μL/mL, 100 μL/mL, 1,000 μL/mL) to plant extract and subsequent analysis by HPLC (Torelli et al., 2000).

##### Abscisic Acid Analysis

The leaf and root extracts were filtered through a 0.2-μm membrane using the filtration syringe system. Samples (20 μL) were injected into a reverse phase C18 HPLC column, and elution was carried out with a mixture of 55% methanol (in 0.1 M acetic acid) using isocratic pump. The eluate was monitored at 260 nm. Standard ABA was used to identify the desired peak in the samples. Method validation was performed by adding known quantities of the standard ABA (10 μL/mL, 100 μL/mL, and 1,000 μL/mL) to the plant extract and subsequent analysis by HPLC (Lim et al., 2005).

##### Brassinosteroids Analysis

1-Napthalenebronic acid (10 mg) was dissolved in dry pyridine (1 mL) and then 100 μL of the solution was added to the sample (both LE and RE). The mixture was heated at 70°C for 30 min. The hot samples were then left to cool for 2 h. When the samples got cold, ether was added for obtaining samples. Samples (20 μL) were injected into a reverse-phase C18 HPLC column and elution was carried out through a mixture of acetonitrile and water with 0.01% H_3_PO_4_ using isocratic pump. The eluate was monitored at 280 nm. Standard BR was used to identify the desired peak in the samples. Method validation was performed by adding known quantities of the standard BR (10 μL/mL, 100 μL/mL, and 1,000 μL/mL) to plant extract and subsequent analysis by HPLC (Swaczynová et al., 2007).

#### Enzymatic Activities

##### Oxidase Activity Determination

A 1 mL reaction mixture for the determination of oxidase activity was prepared by adding 200 μL of sample (RE and LE) into 1.5 mL of phosphate buffer (50 mM), 200 μLH_2_O_2_ (0.1 mM), and 200 μL of ascorbic acid (0.5 mM) in cuvettes. ODs were recorded for each sample for five times with a gap of 30 s at 290 nm. The data so obtained for each sample were averaged and expressed in enzyme units per gram of tested sample (U gm^−1^).

##### Catalase Activity Determination

A sample of LE and RE (40 μL) was added to 2.6 mL of phosphate buffer (50 mM) and 400 μL of H_2_O_2_ (15 mM). OD was recorded at 240 nm for each sample for five times with a gap of 30 s. The data so obtained for each sample were averaged and expressed in enzyme units per gram of tested sample (U gm^−1^).

#### Radical Scavenging Activity

##### 2,2-Diphenyl-1-Picrylhydrazyl Inhibition Assay

Sample (2 mL) of LE and RE was centrifuged at 6,077 rcf for 10 min and then 5 μL of supernatant was added in 3.9 mL of methanolic 2,2-diphenyl-1-picrylhydrazyl (DPPH). The mixture was vigorously shaken and was transferred to a dark place to stand for 30 min. The OD of the sample was observed at 515 nm by a spectrophotometer. Absorbance of DDPH was also noted without adding the sample. Methanolic DDPH was made by dissolving 2.4 g of DPPH in 100 mL of methanol.

The following formula was used to determine the DPPH inhibition.

$$\begin{array}{l} DPPH\;inhibition\;\left(\%\right)=\frac{\left(AB-AA\right)}{AB}\times 100 \end{array}$$

Where, AA is the OD of the sample after 30 min and AB is the OD of the DPPH without sample (Sheng et al., 2011).

##### 2,2′-Azino-bis-3-Ethylbenzothiazoline-6-Sulfonic Acid Inhibition Assay

Potassium persulfate (2.45 mM) and 2,2′-azino-bis-3-ethylbenzothiazoline-6-sulfonic acid (ABTS) (7 mM) were mixed in a ratio of 1:1 (v/v). The mixture was diluted with distilled water till the OD reached to 0.7 at 734 nm. Sample (5 μL) (both LE and RE) was then added to the 3.9 mL of diluted solution and the absorbance was noted.

The following formula was used to determine the ABTS inhibition.

$$\begin{array}{l} ABTS\;inhibition\;\left(\%\right)=\frac{\left(AB-AA\right)}{AB}\times 100 \end{array}$$

Where, AA is the OD of the sample after 30 min and AB is the OD of the ABTS without sample (Sheng et al., 2011).

### Ferric Reducing Antioxidant Power Determination

The ferric reducing antioxidant power (FRAP) reagent was made freshly by mixing 100 μL of 10 mM TPTZ (Sigma Chemical), 1,020 μL of 300 mM sodium acetate (pH 3.6), and 100 μL of 20 mM ferric chloride. The prepared FRAP reagent was then added to 10 μL of a sample (both LE and RE) and the reaction mixture was incubated at 37°C for 60 min. The absorbance was finally read at 593 nm using a UV–vis spectrophotometer after 30 min. Various concentrations of Trolox (10 mg/L, 100 mg/L, and 1,000 mg/L) were used to make a standard curve. Readings of samples were compared with the standard by using straight line equation and the FRAP activity was shown as mg Trolox equivalent/g dw (Fernandes et al., 2016).

### Method for *in-silico* Study

Data of co-expression of auxins signaling gene at the perception level was analyzed using Expression Angler 2016 (http://bar.utoronto.ca/ExpressionAngler/) at BAR keeping the r-cutoff range for upregulation as from 0.7 to 1.0, while for downregulation −0.1 to −0.7. Accession numbers of genes used in the current study are given in Supplementary Table 1. Data extracted were formatted and visualized using yED Graph Editor. In each diagram, red balls were used to define the upregulating genes with the subject gene and blue balls were used to define the downregulating gene. The description of genes was studied on The *Arabidopsis* Information Resource (*TAIR*; www.arabidopsis.org).

### Statistical Analysis

Data obtained were subjected to different statistical procedures, including mean, standard error, ANOVA, and Duncan multiple range test using SPSS for windows 16.0 (SPSS Inc., Chicago, IL, United States). Mean comparison was done by setting reference probability at *p* = 0.05.

## Results

### Growth Kinetics

**Figure 1:**
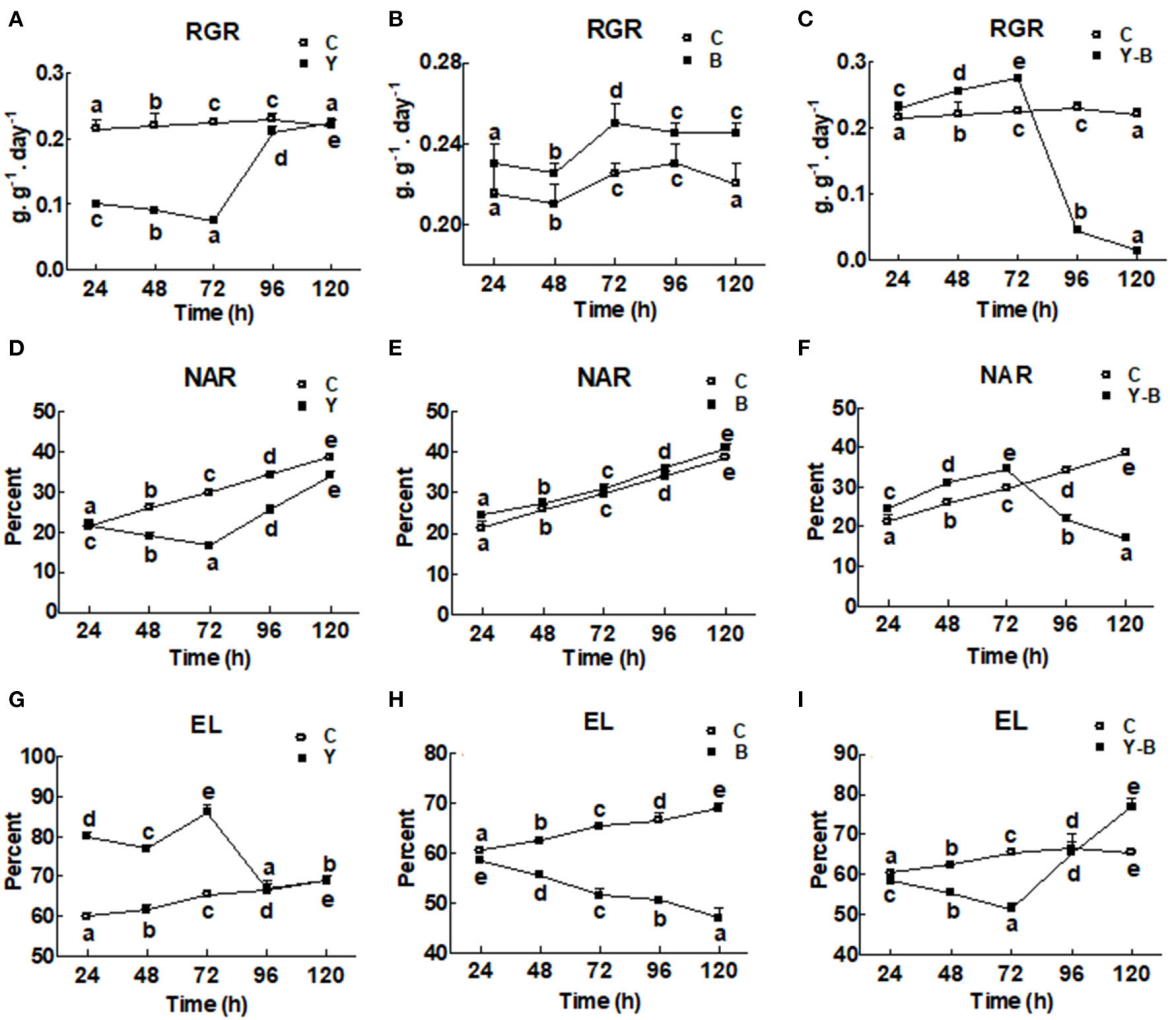
Determination of relative growth rate (RGR; **A–C**), net assimilation rate (NAR; **D–F**), and electrolytic content leakage (EC; **G–I**) in the 11-days-old maize seedlings exposed to different treatments including C (control), Y (yucasin), B (*Bipolaris spp*.), Y-B (yucasin-*Bipolaris* spp.) for the different time duration. Data are mean from three independent experiments with standard error bars. Points on the lines labeled with different letters are significantly different (Duncan test; *p* < 0.05).

The RGR and the NAR were very low in yucasin-treated seedling until 72 h, followed by a steep increase approaching the control at 96 and 120 h (Figures 1A,D). In *Bipolaris*-treated seedlings, both RGR and NAR were higher than the control (Figures 1B,E). In yucasin–*Bipolaris* seedlings, an increasing trend in both RGR and NAR were recorded till 72 h, followed by a sharp decline at 96 and 120 h (Figures 1C,F).

Electrolytic leakage was higher in yucasin seedlings until 72 h, but it became same as control at 96 and 120 h (Figure 1G). In *Bipolaris* seedlings, EL was lower than the control (Figure 1H). Contrary to this, in yucasin–*Bipolaris* seedlings, it was lower till 72 h, but leveled the control at 96 h and significantly increased as compared to the control at 120 h (Figure 1I).

### Fungal Colonization in Root

**Figure 2:**
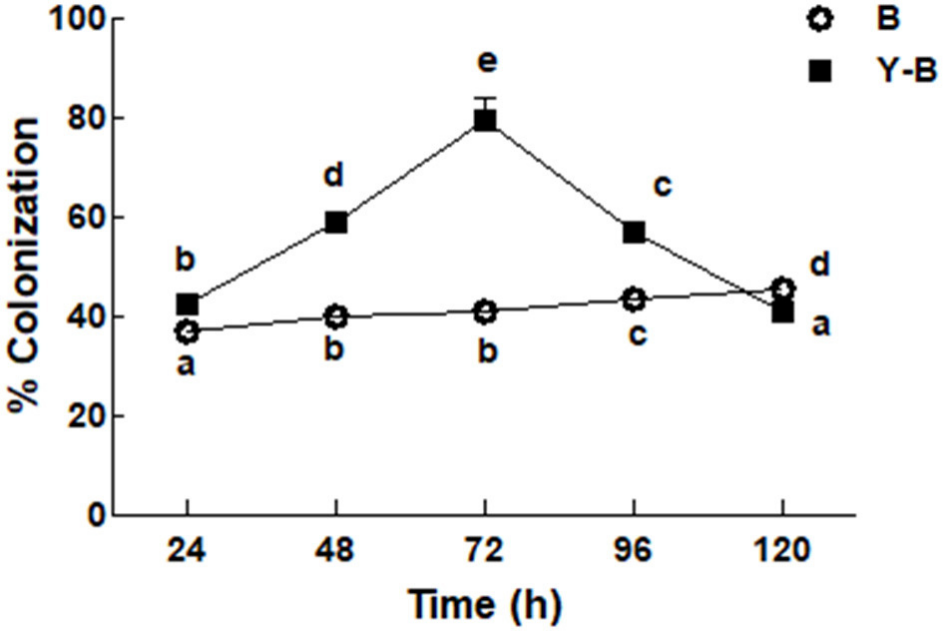
Determination of fungal colonization on maize root under the stress of *B (Bipolaris* spp.), Y–B (yucasin–*Bipolaris* spp.). Data are mean from three independent experiments with standard error bars. Bars labeled with different letters are significantly different (Duncan test; *p* < 0.05).

Fungal colonization was low in the roots of *Bipolaris* seedlings compared to the roots of yucasin–*Bipolaris* seedlings during the experimental time. Contrary to *Bipolaris*, in yucasin–*Bipolaris* seedlings, it was initially very high (72 h), but then decreased with the time, that is, at 96 and 120 h (Figure 2).

### Secondary Metabolites

**Figure 3:**
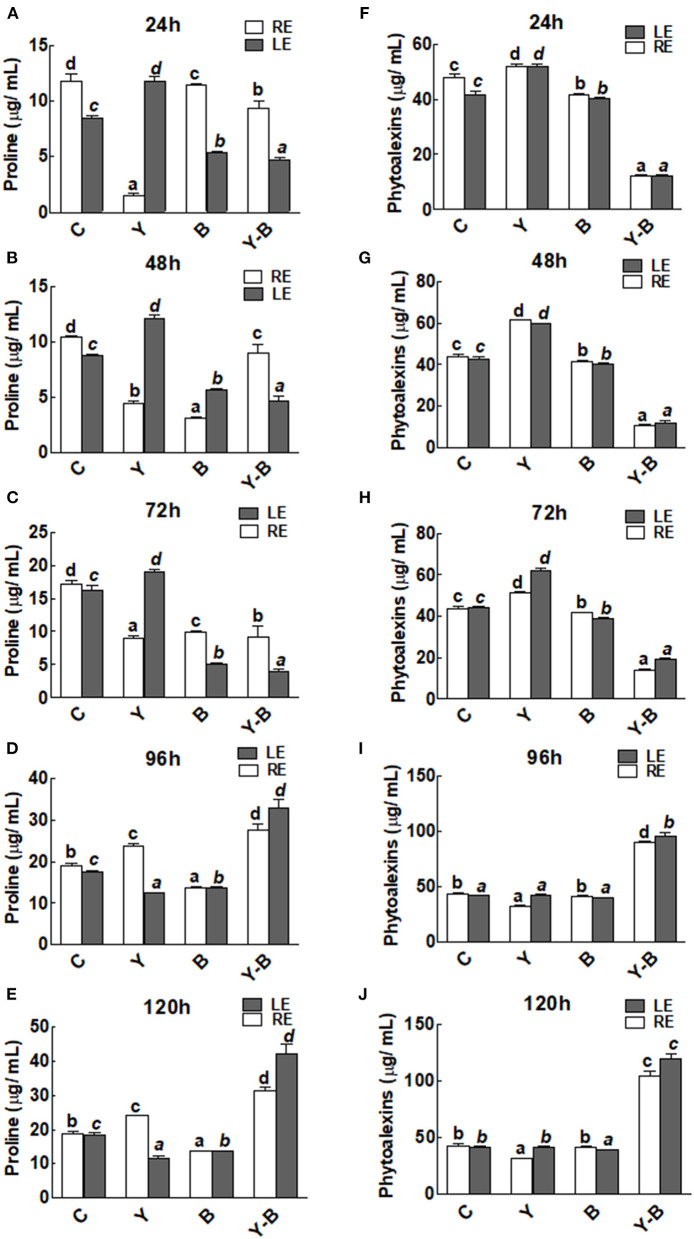
Determination of proline **(A–E)** and phytoalexins **(F–J)** in the leaf extracts (LE) and root exudates (RE) of 11-days-old maize seedlings exposed to different treatments, including C (control); Y (yucasin), B (*Bipolaris* spp.), Y-B (yucasin-*Bipolaris* spp.) for the different time duration. Data are mean from three independent experiments with standard error bars. Bars labeled with different letters are significantly different (Duncan test; *p* < 0.05).

An interesting observation was noticed in the form of reduction in the exudation of proline by yucasin seedlings at the earlier stages of the experiment (72 h). Endogenous level of proline was characterized by the level of proline in leaf (obtained from leaf extract) while the exogenous level of proline was characterized by the level of proline exudated by root (obtained from hydroponic solution). However, in the later hours, proline exudation was significantly increased over the control treatment in roots (Figures 3A–E). On the contrary, the endogenous proline contents of the leaves from yucasin seedlings showed an opposite trend to the root exuded proline. Moreover, *Bipolaris*-associated seedlings had lower amounts of proline than the control both in RE and LE throughout the experimental time. In yucasin–*Bipolaris* seedlings, proline were lower than the control both in RE and LE until 72 h. A dramatic increase was later on recorded in the following hours shooting the amount of proline even higher than the control (Figures 3A–E).

There was no change in the concentration of phytoalexins in both LE and RE of yucasin seedlings. However, it remained comparatively low in *Bipolaris* seedlings. On the contrary, the concentration was very low in yucasin–*Bipolaris* until 72 h. Afterward, the concentration of phytoalexins was shot and touched a peak at 120 h (Figures 3F–J).

### Enzymatic Analysis

**Figure 4:**
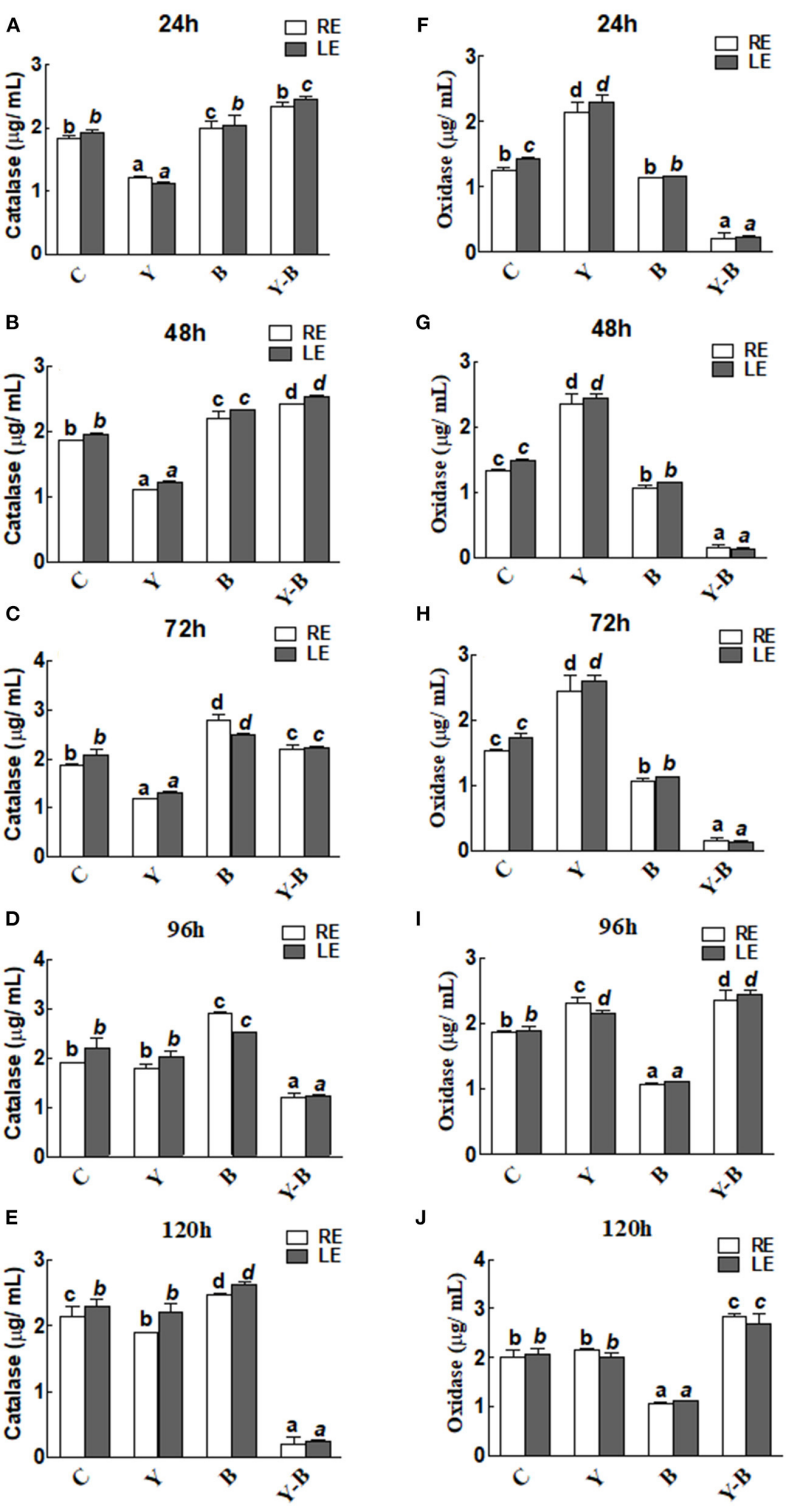
Determination of catalase **(A–E)** and oxidase **(F–J)** activity in the leaf extracts (LE) and root exudates (RE) of 11-days-old maize seedlings exposed to different treatments, including C (control); Y (yucasin), B (*Bipolaris* spp.), Y-B (yucasin-*Bipolaris* spp.) for the different time duration. Data are mean from three independent experiments with standard error bars. Bars labeled with different letters are significantly different (Duncan test; *p* < 0.05).

Catalase and oxidase activities in the RE and LE of the all-treated seedlings were the same. In yucasin seedlings the activity of catalase was low up to 72 h, which then increased and reached same as control in the following hours (Figures 4A–E). Opposingly, a higher activity of catalase was observed in *Bipolaris* seedlings during the whole period of experiment. More interestingly, the activity of catalase was high in yucasin–*Bipolaris* seedlings up to 72 h, while it decreased sharply in the following hours (Figures 4A–E).

As opposed to catalase, the oxidase activity was high in yucasin seedlings until 72 h, but it followed a similar trend as a control in the later hours. In *Bipolaris* seedlings, the activity of the oxidase was low throughout the experiment. Contrary to this, the activity was low in yucasin–*Bipolaris* seedlings at the start, while it increased sharply after 72 h (Figures 4F–J).

### Antioxidant Capacity

**Figure 5:**
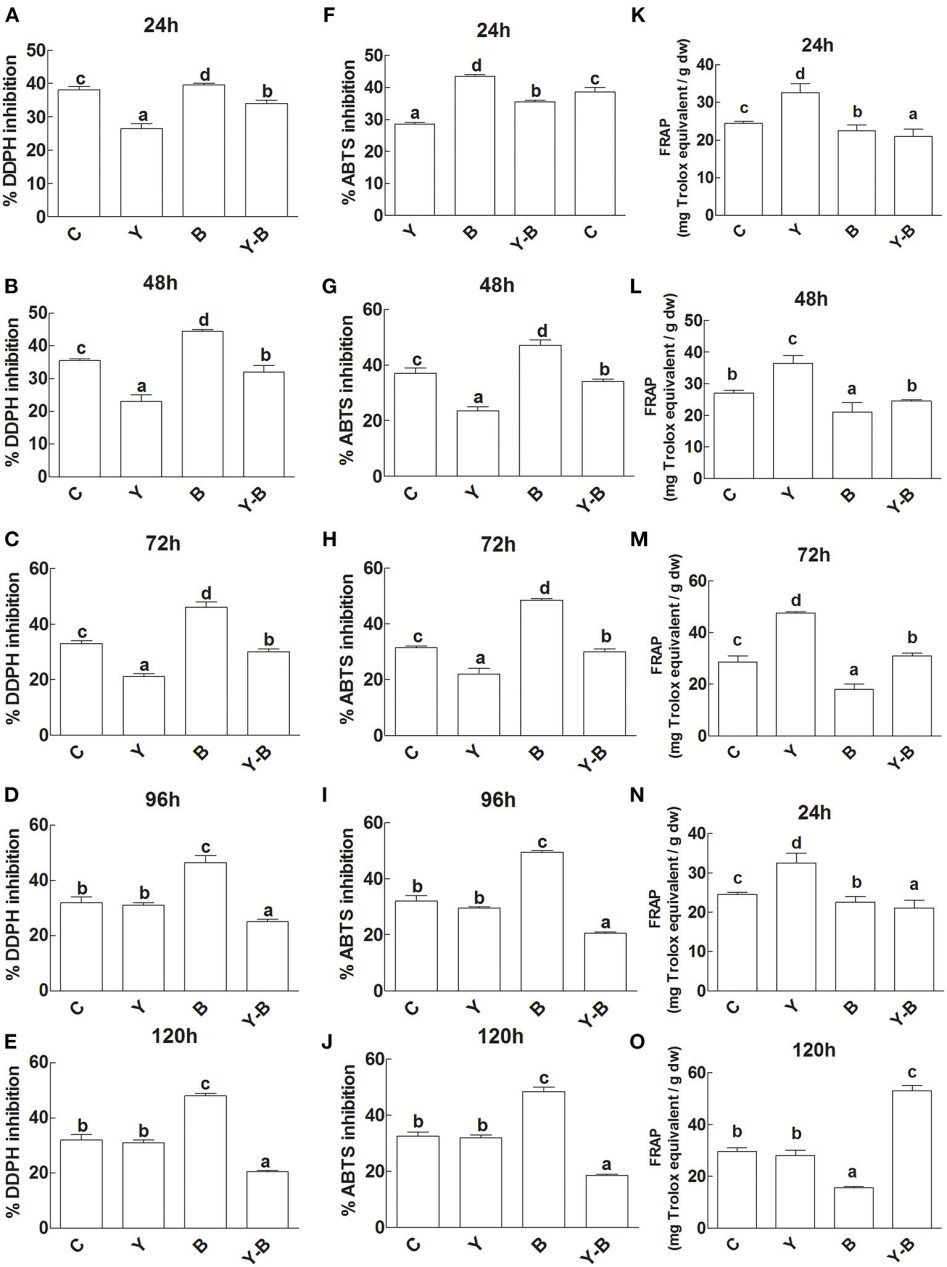
Determination of DDPH inhibition **(A–E)**, ABTS inhibition **(F–J)**, and FRAP activity **(K–O)** in the leaf extracts (LE) and root exudates (RE) of 11-days-old maize seedlings exposed to different treatments including C (control); Y (yucasin), B (*Bipolaris* spp.), Y-B (yucasin-*Bipolaris* spp.) for the different time duration. Data are mean from three independent experiments with standard error bars. Bars labeled with different letters are significantly different (Duncan test; *p* < 0.05).

The antioxidant capacity of the seedlings was determined by DPPH, ABTS inhibition, and FRAPS activity. Initially DPPH and ABTS inhibition was very low in the LE of yucasin seedlings, while in the later hours (120 h) the activity became normal as a control. A high inhibition was noted in the LE of *Bipolaris* seedlings. More effectively, the inhibition was high in yucasin–*Bipolaris* until 72 h, which then declined in the latter part of the experiment (Figures 5A–J).

Unlikely, yucasin seedlings showed reduced FRAP activity at the start of the experiment that acquired a similar trend as a control in the later hours (120 h). Similarly, in *Bipolaris* seedlings, the activity remained low throughout the experiment. Furthermore, the FRAP activity was very low at the initial stages (72 h) of the experiment, which then increased following the latter hours of the experiment (Figures 5K–O).

### Hormonal Analysis

**Figure 6:**
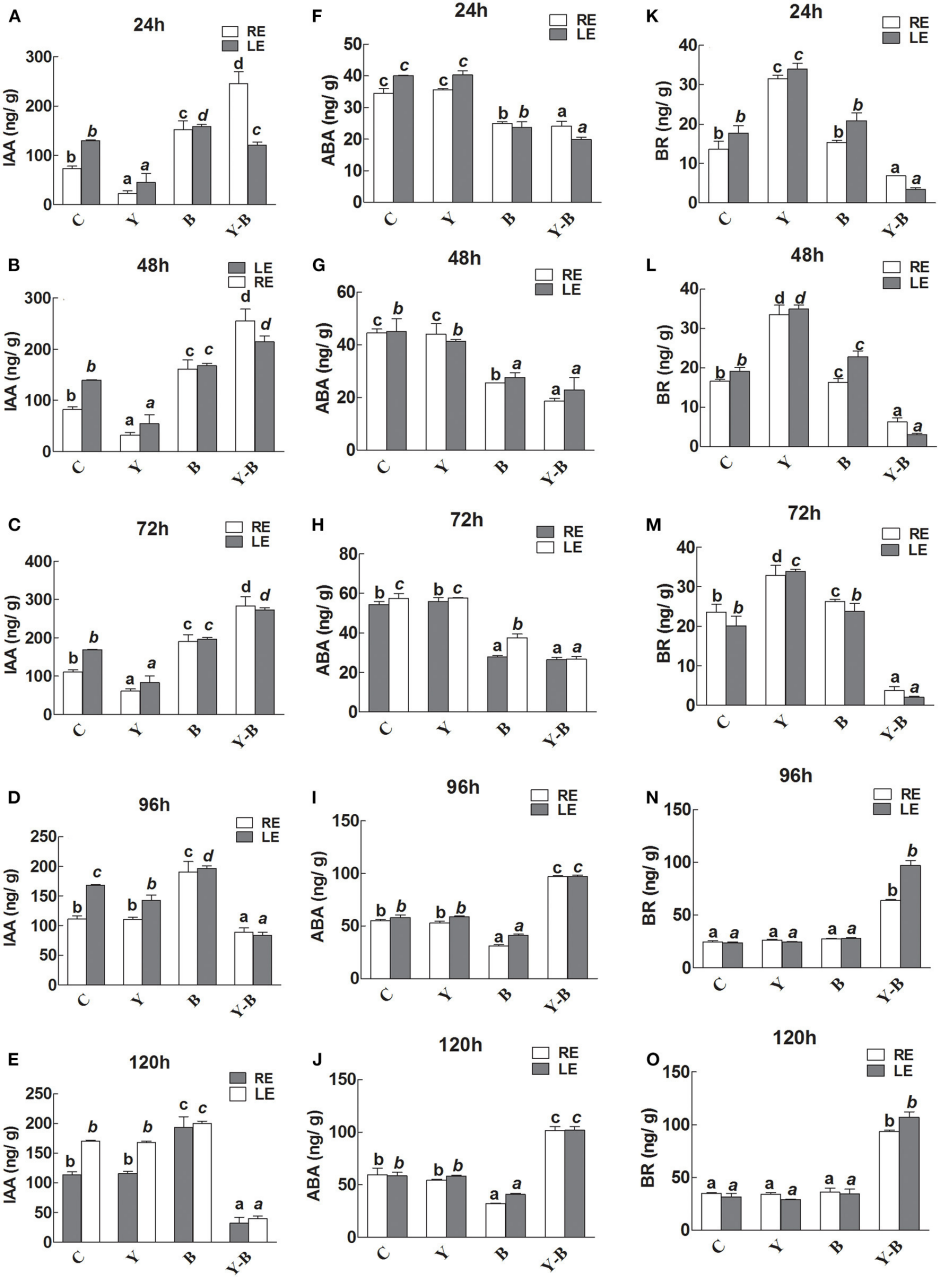
Determination of IAA **(A–E)**, abscisic acid (ABA; **F–J**), and brassinosteroids (BR; **K–O**) in the leaf extracts (LE) and root exudates (RE) of 11-days-old maize seedlings exposed to different treatments, including C (control); Y (yucasin), B (*Bipolaris* spp.), Y-B (yucasin-*Bipolaris* spp.) for the different time duration. Data are mean from three independent experiments with standard error bars. Bars labeled with different letters are significantly different (Duncan test; *p* < 0.05).

A lower level of IAA was noted in RE and LE of yucasin seedlings until 72 h. After passing the 72 h, the IAA level was retained. Additionally, the IAA level was slightly high in LE and RE of *Bipolaris* seedlings during the whole experimental period (Figures 6A–E). The IAA levels in RE and LE of yucasin–*Bipolaris* seedlings was observed to be increased until 72 h, which then decreased with the passage of time and reached to a low value at 120 h.

There was no effect of yucasin on ABA concentration as it remained same as control, whereas the concentration was slightly low in *Bipolaris* seedlings. The ABA level in yucasin–*Bipolaris* seedlings was significantly lower than control until 72 h that increased in the later hours of the experiment (Figures 6F–J).

In yucasin seedlings, the BR level was increased until 72 h, which then decreased in the later hours, whereas it was slightly high in *Bipolaris* seedlings. In yucasin–*Bipolaris* seedlings, the level was extremely low until 72 h. After 72 h, the level abruptly increased and reached to a peak value at the end of the experiment (120 h) (Figures 6K–O).

### Co-Expression at Auxin Reception

**Figure 7:**
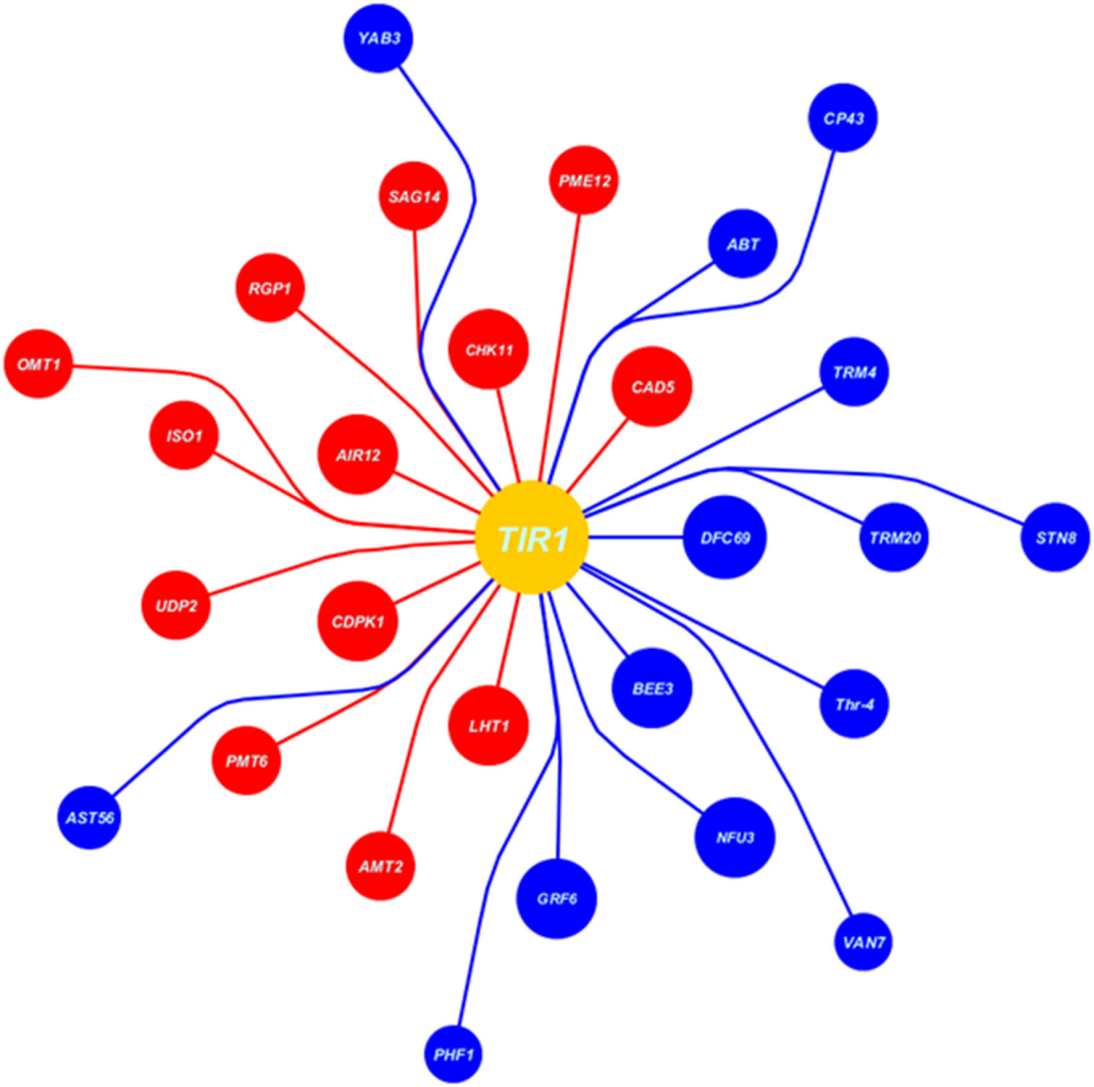
Data extracted from Expression Angler (2016) showing the set of co-expressed genes with *TIR1* (auxin signaling gene) under biotrophic stress (*Erysiphe orontii*) as blue balls indicates the downregulated genes, while red indicates the upregulated genes. Moreover, the area of the ball shows the *R*-value of co-expressed gene, larger the *R*-value larger the area of the ball and nearer to the *TIR1* ball.

**Figure 8:**
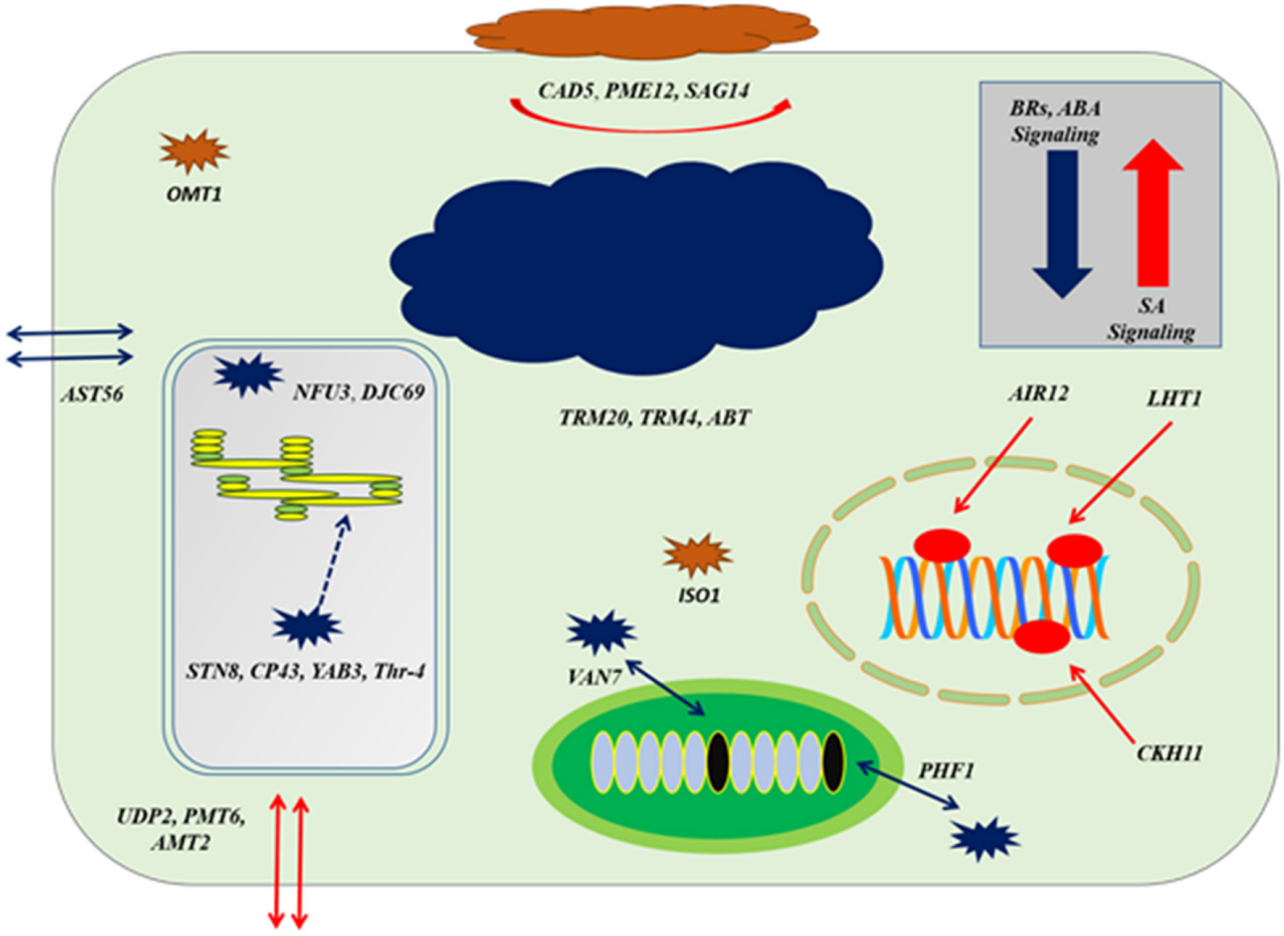
The cellular response at genes co-expression of auxins signaling gene (*TIR1*) based on Figure 7; at the level of upregulation *TIR1*, deposition of cell wall materials of upregulated genes, such as *CAD5, PME12*, and *SAG14* with the cell-specific transporters, such as *UDP2, PMT6*, and *AMT2* increased. The enzymatic reactions of flavonoids degradation (*ISO1*) and ROS productions (*OMT1*) were also increased proliferating microbial growth. During downregulation of *TIR1*, genes regulating photosynthetic rate and thylakoid organization, such *STN8, CP43, YAB3*, and *Thr-4*, VAN7, and PHF1 for mitochondrial organization and *TRM20, TRM4*, and *ABT* were downregulated, deterring cellular growth. Regarding other phytohormonal signaling, ABA and BR signaling systems were reduced while SA signaling was induced.

At auxin reception in host plant during high auxin levels, the cysteine signaling (*LHT1*), ABA inhibiting signaling (*CDPK1*), IAA signaling in root (*AIR12*), and receptor-like protein kinase3 (*CHK11*) were upregulated that deterred the defense-mediated response in the host against *Erysiphe orontii* (a biotrophic pathogen) (Figures 7, 8). *CAD5-, PME12-*, and *SAG14-encoded enzymes* catalyzed the group of precursors, including sinapyl alcohol, caffeyl alcohol, coniferyl alcohol, d-hydroxyconiferyl, and p-coumaryl aldehyde to lignin and its deposition in the host cell wall. Similarly, glycosylated polypeptide 1 (*RGP1*) catalyzed the pyranose and furanose into UDP-L-arabinose for cell wall modification against necrotrophic pathogen. The myristoylation of many signaling proteins and encoding of flavonol-3-*O*-methyl transferase (*OMT1*) played a vital role to inhibit the production as well as modification of secondary metabolites (flavonoids, myricetin). Other processes, such as starch biosynthesis (*ISO1*), galactose transporter (*UDP2*), polyol transporter (*PMT6*), and ammonium transporter (*AMT2*) were upregulated against biotrophic microbe proliferation (Figures 7, 8).

Downregulation at auxin reception with IAA-compromised host, included upregulation of BR biosynthesis and their signaling system by *BEE3* and *GRF6*, respectively. Chloroplast-localized chaperon proteins (*NFU3*) and (*DJC69*) as well as chloroplast thylakoid–signaling proteins (*STN8*), (*CP43*), (*YAB3*), and (Thr-4), controlling the stability of photosystem II were downregulated deterring the photosynthetic rate. Downregulation of enzymes, such as ethyl-coenzyme M reductase II subunit gamma (*TRM20*) (TRM4), oligogalacturonide oxidase (*ABT*) resulted in the destabilization of the host cell, which is not in favor of biotrophic pathogen. Transportation of potassium, sulfur, and other nutrients by transmembrane factors (*AST56*), (*PHF1)*, and (*VAN7)* were downregulated to limit the supply of nutrients to biotrophs at the very extreme stage of virulent (Figures 7, 8).

## Discussion

Auxin is primarily responsible for many morphological and physiological processes, such as fruit development, shoot and root architecture, cell elongation, tropisms, and tissue differentiation (Grones et al., 2015). Besides, auxin biosynthesis, signaling and inactivation are correlated with plant microbial interactions (Maag et al., 2015). In our analysis, the endogenous IAA dropped significantly as a result of yucasin treatment during first 72 h of exposure. Low IAA phenotype was characterized by compromised rate of photosynthesis and growth, which were recorded as NAR and RGR, respectively. However, after 72 h of yucasin exposure, the effect was neutralized and IAA level was restored, subsequently improved the RGR and NAR. NAR and RGR are regarded as the basic parameters to determine the growth of plants/crops under abiotic and biotic stress (Rajput et al., 2017).

Another important observation was compromised membrane stability in yucasin-treated seedlings as indicated by high leakage of electrolytes until 72 h post-treatment. However, EL was significantly reduced as a result of increase in the level of endogenous IAA during later hours. Genetic studies revealed that yucasin acts as a limiting rate enzyme in the biosynthesis of IAA. Therefore, *YUC* gene plays a crucial role in the regulation of many physiological and developmental processes (Suzuki et al., 2016). *Bipolaris* spp. was used as a growth-promoting agent because of its high colonization activity and secretion of essential biomolecules, deduced from the optimal growth condition of *Bipolaris* seedlings and root exudation. When this endophyte was inoculated on low IAA seedlings (yucasin treated), the level of IAA was shot that resulted in the prolific fungal colonization of maize roots (72%) in 72 h. Fungal colonization was positively correlated with the growth seedling. In earlier studies, IAA has been implicated in shaping plant–microbe interaction enabling fungal endophyte to successfully colonize host plant tissues (Mehmood et al., 2020). During the current study, we found that due to prolific colonization of maize seedlings by *Bipolaris*, IAA was drastically increased in host tissues after 72 h of fungal exposure, inhibiting host growth. It has been known that plant can rapidly and reversibly modulate growth in response to addition or removal of IAA. For instance, *Arabidopsis* showed a rapid decline in growth when its roots were exposed to nanomolar concentration of IAA (Fendrych et al., 2018). Plant pathogens stimulate host susceptibility by manipulating the auxin levels of the host, its transport and signaling (Naseem et al., 2015). In this context, *Pseudomonas syringae* tomato is worth mentioning, which is known to promote host susceptibility by increasing the auxin–cytokinin ratio in the host (Naseem and Dandekar, 2012). Under stress conditions, such as high salinity or any pathogenic attack, EL is characterized by mainly K^+^ exudation that solely reveals the accumulation of reactive oxygen species (ROS) and subsequent cell death (Demidchik et al., 2014). EL was high in yucasin seedlings due to the accumulation of antioxidant compounds to repair the damaged cells. In contrast, in yucasin–*Bipolaris*, EL was initially very low as high IAA level suppressed the accumulation of antioxidant compounds, thereby inducing prolific seedling growth. In later hours, when IAA level dropped in yucasin–*Bipolaris* seedlings, EL increased deducing the accumulation of antioxidant compounds for cell repairing processes. EL also indicates the stability of plasma membrane for its role in activation of GORK, SKOR, and annexins for K^+^ efflux (Demidchik et al., 2014). During initial hours, the integrity of plasma membrane was sustained in yucasin–*Bipolaris* seedling, while latterly the stability was highly affected due to low IAA level and high fungal colonization. Indole-3-acetic acid is known to affect plasma membrane by changing packing, stability, and interactions between membrane lipids in a concentration-dependant manner (Hac-Wydro and Flasiński, 2015).

Moreover, proline level significantly declined in root exudates upon Y treatment until 72 h of treatment and inclined in the leaf. This trend was reversed at 96 h of the treatment. Proline level plays an important part to alleviate the stress condition in plants and are produced in higher concentrations upon exposure to abiotic stress (Dikilitas et al., 2020). In such circumstances, lower exudation of prolin might be a wise strategy to conserve endogenous prolin pool. In later hours, excess of prolin accumulated was exuded through root. In yucasin-treated seedlings, phytoalexins remained higher than the control until 72 h post-treatment and dropped rapidly in later hours. Apparently, yucasin has induced stress in maize seedlings, which lasted for 72 h and plants respond to stress by accumulating higher concentrations of phytoalexins (Jeandet, 2015). Later on when the effect of stress vanished, concentration of phytoalexins dropped to the level comparable to the control. However, in binary combination with *Bipolaris*, an exactly opposite trend was recorded for phytoalexins. *Bipolaris* efficiently minimized yucasin-induced stress in maize initially, but after 96 h of inoculation, it became a growth inhibitor which might have elevated the levels of phytoalexins in the host seedlings (Jeandet, 2015).

Enzymatic activities such as catalase and oxidase activities were also analyzed under the treatments. It has been found that catalase and oxidase activities were operating antagonistically to each other. In previous studies, it was noticed that catalase activity in the seedlings decreased under stress and at the same time oxidase activity was increased. Similarly, in our studies, it is elucidated that the time in which seedlings were in stress, the catalase activity was low while the oxidase activity was high (Demircan et al., 2020).

The increase in IAA up to the highest level in root exudates might be due to fungal colonization, while endogenous level of IAA in leaf might be the seedling factor. Previous studies indicated that elevated level of IAA in plant was because of microbial infection (Zhang et al., 2018). Microbial application to the host increased the *YUC* and *TIR1* transcripts in host plants, which simultaneously increased the endogenous level of IAA, turning the endophytic fungus to apathogenic one (Wang and Fu, 2011). Interestingly, in the later hours, all the observations were reversed as the seedlings compromised their immunity and the endophytic fungus has switched to pathogenic fungi. At this condition, yucasin–*Bipolaris* seedlings increased the BRs and phytoalexins level, which was very low during high IAA level in the host leaf. BRs perform a versatile role in the context of hormonal cross-talk under plant microbial interaction through its biosynthesis and binding with BR1 (Alazem and Lin, 2015).

BRs increase host resistance through RBOHB-dependent ROS burst and MEK2-SIPK cascade. Likewise, BES1/BZR1 repressed RBOHB-dependent ROS production and acted as an important mediator of the substitution between immunity and growth in BR signaling (Fonseca et al., 2018). Similarly, high phytoalexins level also decreased growth with the expense of host immunity. In this connection, it is important that the seedlings tried to cope with the oxidative burst of BRs by producing higher quantities of proline and other antioxidant compounds. Therefore, the ABTS, DPPH inhibition activity was quite low in yucasin–*Bipolaris* seedling after 72 h, which was initially high. Similarly, FRAP antioxidant activity of yucasin–*Bipolaris* seedling was high after 72 h, which was very low at the start. During pathogenic attack, the host produces a specific set of ROS known as ROS signature that elicits a desired photochemical and physiological response related to defense (De Gara et al., 2003).

Through *in-silico* analysis (auxins signaling during plant microbial interaction), it was observed that during a high IAA level, the plant compromised its immunity by inducing high growth. This was evident from myristylation and encoding of flavonol-3-methyltransferase, which inhibited the production of secondary metabolites to promote growth response. During a high IAA level, production of antioxidant and other secondary metabolites decreased that led to plant growth promotion (Cruz et al., 2018). That is why the radical scavenging activity was very low in yucasin–*Bipolaris* seedlings at the start of the experiment; thus, plant growth promotion was achieved. Moreover, at a high IAA level, biosynthesis and transport of starch and transportation of polyol and ammonium across the cell membrane mediated microbial growth. This deduced the high *Bipolaris* colonization on root. Fungal colonization on maize seedlings requires high efflux of starch and ammonium ions across the apoplast for its nourishment through AMTs transport family proteins (Ariz et al., 2018). Moreover, *in-silico* analysis, high IAA level also interfered with the co-related cell defense signaling, such as CDPK1, ABA inhibiting, and IAA signaling in root that further increased the overgrowth of microbe.

Noticeably, at a high IAA level, the downregulation of BRs signaling and biosynthesis weaken the host cells against virulence. Our results are in line with a previous study, where high IAA level inferred with BR signaling switching plant defense response against various pathogens (Rasool et al., 2018). That is why, IAA at extreme level in maize seedlings played antagonistic role in BR biosynthesis. Similarly, under the optimal level of IAA, encoding of the ethyl-coenzyme M reductase II subunit gamma and oligogalacturonide oxidase were found to be increased under IAA-ABA and BR homeostasis, aided the host immunity. However, the system was negatively interrupted at a high level of IAA.

## Conclusions

Auxin is the group of signaling molecules that play a vital role in plant–microbial interaction. IAA is mainly produced in maize seedlings through yucasin pathway. Under yucasin stress to the seedlings, IAA was inhibited, which decreased seedling growth. After inoculation of *Bipolaris*, the seedlings retained their growth initially, but in later hours, the growth was highly compromised due to high fungal colonization rate at high IAA levels. Low growth of yucasin–*Bipolaris* seedlings further reinforced by the antagonistic effect of high IAA with BR and phytoalexins that adversely affected the seedling defense response.

## Data Availability Statement

The raw data supporting the conclusions of this article will be made available by the authors, without undue reservation.

## Author Contributions

MY performed the manipulations and wrote draft manuscript. AH supervised the research work and proposed the idea. MH supervised bioinformatic work. AI improved the draft manuscript. MI carried out some experimental procedures. H-YK provided research facilities and guideline. I-JL supported the research work by providing resources. All authors contributed to the article and approved the submitted version.

## Conflict of Interest

The authors declare that the research was conducted in the absence of any commercial or financial relationships that could be construed as a potential conflict of interest.

## Publisher's Note

All claims expressed in this article are solely those of the authors and do not necessarily represent those of their affiliated organizations, or those of the publisher, the editors and the reviewers. Any product that may be evaluated in this article, or claim that may be made by its manufacturer, is not guaranteed or endorsed by the publisher.
