# Supplementary material for: Transformation of Endophytic Bipolaris spp. Into Biotrophic Pathogen Under Auxin Cross-Talk With Brassinosteroids and Abscisic Acid
Source: Front Bioeng Biotechnol. 2021 Jul 28;9:657635. doi: 10.3389/fbioe.2021.657635 (PMC8355742; doi:10.3389/fbioe.2021.657635)
Supplement: Supplementary file 1 [file Table_1.DOCX]

**Accession Numbers of Genes used during current study**

Data of gene expression was obtained from eFP browser ([Arabidopsis eFP Browser 2.0 (utoronto.ca)](http://bar.utoronto.ca/efp2/Arabidopsis/Arabidopsis_eFPBrowser2.html)) which is verified through rtPCR by the database.

| **S. No.** | **Gene** | **Accession Number** |
| --- | --- | --- |
|  | *TIR1* | AT3G62980 |
|  | *CAD5* | AT4G34230 |
|  | *PME12* | AT2G26440 |
|  | *SAG14* | AT5G20230 |
|  | *UDP2* | AT1G02000 |
|  | *PMT6* | AT4G36670 |
|  | *AMT2* | AT2G38290 |
|  | *ISO1* | AT1G03310 |
|  | *OMT1* | AT5G54160 |
|  | *STN8* | AT5G01920 |
|  | *CP43* | ATCG00280 |
|  | *YAB3* | AT4G00180 |
|  | *Thr-4* | AT5G01920 |
|  | *VAN7* | AT1G13980 |
|  | *PHF1* | AT3G52190 |
|  | *TRM20* | AT4G28760 |
|  | *TRM4* | AT1G74160 |
|  | *ABT* | AT4G08870 |
|  | *DFC69* | AT3G10160 |
|  | *CHK11* | AT3G05330 |
|  | *RGP1* | AT3G02230 |
|  | *AIR12* | AT3G07390 |
|  | *CDPK1* | AT1G18890 |
|  | *LHT1* | AT5G40780 |
|  | *BEE3* | AT1G73830 |
|  | *NFU3* | AT4G25910 |
|  | *GRF6* | AT2G06200 |
|  | *AST56* | AT1G77990 |
